# Supplementary material for: Pyruvate Dehydrogenase Kinase 1 inhibition mediated oxidative phosphorylation enhancement in cartilage promotes osteoarthritis progression
Source: BMC Musculoskelet Disord. 2023 Jul 20;24:597. doi: 10.1186/s12891-023-06585-6 (PMC10357736; doi:10.1186/s12891-023-06585-6)
Supplement: Supplementary file 1 — Additional file 1. [file 12891_2023_6585_MOESM1_ESM.zip › BP.Gsea.1653623667859/GOBP_SIGNAL_TRANSDUCTION_BY_P53_CLASS_MEDIATOR.html]

Details for gene set GOBP\_SIGNAL\_TRANSDUCTION\_BY\_P53\_CLASS\_MEDIATOR[GSEA]

|  || Dataset | 55457\_collapsed\_to\_symbols.cls.cls #OA\_versus\_normal.cls.cls #OA\_versus\_normal\_repos |
| Phenotype | cls.cls#OA\_versus\_normal\_repos |
| Upregulated in class | normal |
| GeneSet | GOBP\_SIGNAL\_TRANSDUCTION\_BY\_P53\_CLASS\_MEDIATOR |
| Enrichment Score (ES) | -0.3718257 |
| Normalized Enrichment Score (NES) | -1.7067629 |
| Nominal p-value | 0.0 |
| FDR q-value | 0.12910484 |
| FWER p-Value | 1.0 |
Table: GSEA Results Summary

  

Fig 1: Enrichment plot: GOBP\_SIGNAL\_TRANSDUCTION\_BY\_P53\_CLASS\_MEDIATOR      
 Profile of the Running ES Score & Positions of GeneSet Members on the Rank Ordered List

  

| SYMBOL | TITLE | RANK IN GENE LIST | RANK METRIC SCORE | RUNNING ES | CORE ENRICHMENT || 1 | PSMD10 | "proteasome 26S subunit, non-ATPase 10 [Source:HGNC Symbol;Acc:HGNC:9555]" | 58 | 0.976 | 0.0152 | No |
| 2 | HINT1 | histidine triad nucleotide binding protein 1 [Source:HGNC Symbol;Acc:HGNC:4912] | 369 | 0.718 | 0.0054 | No |
| 3 | ZMPSTE24 | zinc metallopeptidase STE24 [Source:HGNC Symbol;Acc:HGNC:12877] | 451 | 0.682 | 0.0129 | No |
| 4 | MIF | macrophage migration inhibitory factor [Source:HGNC Symbol;Acc:HGNC:7097] | 555 | 0.640 | 0.0178 | No |
| 5 | ING4 | inhibitor of growth family member 4 [Source:HGNC Symbol;Acc:HGNC:19423] | 680 | 0.598 | 0.0201 | No |
| 6 | RNF34 | ring finger protein 34 [Source:HGNC Symbol;Acc:HGNC:17297] | 699 | 0.594 | 0.0308 | No |
| 7 | SMYD2 | SET and MYND domain containing 2 [Source:HGNC Symbol;Acc:HGNC:20982] | 856 | 0.553 | 0.0297 | No |
| 8 | PYCARD | PYD and CARD domain containing [Source:HGNC Symbol;Acc:HGNC:16608] | 1164 | 0.484 | 0.0154 | No |
| 9 | AKT1 | AKT serine/threonine kinase 1 [Source:HGNC Symbol;Acc:HGNC:391] | 1192 | 0.478 | 0.0230 | No |
| 10 | GREM1 | "gremlin 1, DAN family BMP antagonist [Source:HGNC Symbol;Acc:HGNC:2001]" | 1255 | 0.467 | 0.0276 | No |
| 11 | AURKB | aurora kinase B [Source:HGNC Symbol;Acc:HGNC:11390] | 1407 | 0.442 | 0.0247 | No |
| 12 | WWOX | WW domain containing oxidoreductase [Source:HGNC Symbol;Acc:HGNC:12799] | 1614 | 0.411 | 0.0169 | No |
| 13 | RRP8 | ribosomal RNA processing 8 [Source:HGNC Symbol;Acc:HGNC:29030] | 1673 | 0.402 | 0.0205 | No |
| 14 | PDK2 | pyruvate dehydrogenase kinase 2 [Source:HGNC Symbol;Acc:HGNC:8810] | 1772 | 0.388 | 0.0206 | No |
| 15 | YJU2 | YJU2 splicing factor homolog [Source:HGNC Symbol;Acc:HGNC:25518] | 1832 | 0.380 | 0.0237 | No |
| 16 | PRKN | parkin RBR E3 ubiquitin protein ligase [Source:HGNC Symbol;Acc:HGNC:8607] | 1972 | 0.364 | 0.0202 | No |
| 17 | PTTG1IP | PTTG1 interacting protein [Source:HGNC Symbol;Acc:HGNC:13524] | 2012 | 0.360 | 0.0244 | No |
| 18 | TAF9B | TATA-box binding protein associated factor 9b [Source:HGNC Symbol;Acc:HGNC:17306] | 2081 | 0.352 | 0.0262 | No |
| 19 | COPS3 | COP9 signalosome subunit 3 [Source:HGNC Symbol;Acc:HGNC:2239] | 2144 | 0.345 | 0.0283 | No |
| 20 | TRIAP1 | TP53 regulated inhibitor of apoptosis 1 [Source:HGNC Symbol;Acc:HGNC:26937] | 2163 | 0.342 | 0.0338 | No |
| 21 | CASP6 | caspase 6 [Source:HGNC Symbol;Acc:HGNC:1507] | 2427 | 0.311 | 0.0195 | No |
| 22 | CHEK2 | checkpoint kinase 2 [Source:HGNC Symbol;Acc:HGNC:16627] | 2533 | 0.299 | 0.0173 | No |
| 23 | FHIT | fragile histidine triad diadenosine triphosphatase [Source:HGNC Symbol;Acc:HGNC:3701] | 2758 | 0.274 | 0.0053 | No |
| 24 | MAGEA2 | MAGE family member A2 [Source:HGNC Symbol;Acc:HGNC:6800] | 2952 | 0.255 | -0.0047 | No |
| 25 | CDKN1B | cyclin dependent kinase inhibitor 1B [Source:HGNC Symbol;Acc:HGNC:1785] | 2984 | 0.251 | -0.0020 | No |
| 26 | CHEK1 | checkpoint kinase 1 [Source:HGNC Symbol;Acc:HGNC:1925] | 3173 | 0.233 | -0.0120 | No |
| 27 | TOPORS | "TOP1 binding arginine/serine rich protein, E3 ubiquitin ligase [Source:HGNC Symbol;Acc:HGNC:21653]" | 3244 | 0.227 | -0.0129 | No |
| 28 | CDIP1 | cell death inducing p53 target 1 [Source:HGNC Symbol;Acc:HGNC:13234] | 3285 | 0.222 | -0.0116 | No |
| 29 | GML | glycosylphosphatidylinositol anchored molecule like [Source:HGNC Symbol;Acc:HGNC:4375] | 3598 | 0.194 | -0.0321 | No |
| 30 | CRADD | CASP2 and RIPK1 domain containing adaptor with death domain [Source:HGNC Symbol;Acc:HGNC:2340] | 3651 | 0.190 | -0.0323 | No |
| 31 | BRCA2 | BRCA2 DNA repair associated [Source:HGNC Symbol;Acc:HGNC:1101] | 3853 | 0.170 | -0.0447 | No |
| 32 | RPS27L | ribosomal protein S27 like [Source:HGNC Symbol;Acc:HGNC:18476] | 3882 | 0.166 | -0.0435 | No |
| 33 | MLF1 | myeloid leukemia factor 1 [Source:HGNC Symbol;Acc:HGNC:7125] | 3971 | 0.158 | -0.0472 | No |
| 34 | NBN | nibrin [Source:HGNC Symbol;Acc:HGNC:7652] | 4136 | 0.143 | -0.0571 | No |
| 35 | PPM1D | "protein phosphatase, Mg2+/Mn2+ dependent 1D [Source:HGNC Symbol;Acc:HGNC:9277]" | 4159 | 0.142 | -0.0560 | No |
| 36 | TMEM109 | transmembrane protein 109 [Source:HGNC Symbol;Acc:HGNC:28771] | 4386 | 0.122 | -0.0712 | No |
| 37 | FOXM1 | forkhead box M1 [Source:HGNC Symbol;Acc:HGNC:3818] | 4741 | 0.092 | -0.0971 | No |
| 38 | ELL3 | elongation factor for RNA polymerase II 3 [Source:HGNC Symbol;Acc:HGNC:23113] | 4801 | 0.087 | -0.1000 | No |
| 39 | AURKA | aurora kinase A [Source:HGNC Symbol;Acc:HGNC:11393] | 4916 | 0.079 | -0.1074 | No |
| 40 | ATAD5 | ATPase family AAA domain containing 5 [Source:HGNC Symbol;Acc:HGNC:25752] | 5020 | 0.071 | -0.1140 | No |
| 41 | BAG6 | BAG cochaperone 6 [Source:HGNC Symbol;Acc:HGNC:13919] | 5023 | 0.071 | -0.1127 | No |
| 42 | E2F1 | E2F transcription factor 1 [Source:HGNC Symbol;Acc:HGNC:3113] | 5137 | 0.061 | -0.1203 | No |
| 43 | SPRED2 | sprouty related EVH1 domain containing 2 [Source:HGNC Symbol;Acc:HGNC:17722] | 5179 | 0.058 | -0.1224 | No |
| 44 | TP73 | tumor protein p73 [Source:HGNC Symbol;Acc:HGNC:12003] | 5222 | 0.055 | -0.1246 | No |
| 45 | DYRK2 | dual specificity tyrosine phosphorylation regulated kinase 2 [Source:HGNC Symbol;Acc:HGNC:3093] | 5224 | 0.054 | -0.1235 | No |
| 46 | SNW1 | SNW domain containing 1 [Source:HGNC Symbol;Acc:HGNC:16696] | 5472 | 0.035 | -0.1422 | No |
| 47 | TP53 | tumor protein p53 [Source:HGNC Symbol;Acc:HGNC:11998] | 5511 | 0.032 | -0.1445 | No |
| 48 | BCL2 | BCL2 apoptosis regulator [Source:HGNC Symbol;Acc:HGNC:990] | 5609 | 0.023 | -0.1517 | No |
| 49 | NUPR1 | "nuclear protein 1, transcriptional regulator [Source:HGNC Symbol;Acc:HGNC:29990]" | 5616 | 0.023 | -0.1517 | No |
| 50 | KAT6A | lysine acetyltransferase 6A [Source:HGNC Symbol;Acc:HGNC:13013] | 5778 | 0.010 | -0.1641 | No |
| 51 | SP100 | SP100 nuclear antigen [Source:HGNC Symbol;Acc:HGNC:11206] | 5899 | 0.002 | -0.1735 | No |
| 52 | MAPKAPK5 | MAPK activated protein kinase 5 [Source:HGNC Symbol;Acc:HGNC:6889] | 6094 | -0.012 | -0.1884 | No |
| 53 | ING2 | inhibitor of growth family member 2 [Source:HGNC Symbol;Acc:HGNC:6063] | 6150 | -0.016 | -0.1924 | No |
| 54 | ATM | ATM serine/threonine kinase [Source:HGNC Symbol;Acc:HGNC:795] | 6179 | -0.019 | -0.1942 | No |
| 55 | PHLDA3 | pleckstrin homology like domain family A member 3 [Source:HGNC Symbol;Acc:HGNC:8934] | 6255 | -0.024 | -0.1996 | No |
| 56 | TP63 | tumor protein p63 [Source:HGNC Symbol;Acc:HGNC:15979] | 6274 | -0.025 | -0.2005 | No |
| 57 | BDKRB2 | bradykinin receptor B2 [Source:HGNC Symbol;Acc:HGNC:1030] | 6276 | -0.026 | -0.2001 | No |
| 58 | EEF1E1 | eukaryotic translation elongation factor 1 epsilon 1 [Source:HGNC Symbol;Acc:HGNC:3212] | 6296 | -0.027 | -0.2010 | No |
| 59 | TAF9 | TATA-box binding protein associated factor 9 [Source:HGNC Symbol;Acc:HGNC:11542] | 6297 | -0.027 | -0.2005 | No |
| 60 | PERP | p53 apoptosis effector related to PMP22 [Source:HGNC Symbol;Acc:HGNC:17637] | 6324 | -0.030 | -0.2019 | No |
| 61 | NOP53 | NOP53 ribosome biogenesis factor [Source:HGNC Symbol;Acc:HGNC:4333] | 6334 | -0.031 | -0.2020 | No |
| 62 | MARCHF7 | membrane associated ring-CH-type finger 7 [Source:HGNC Symbol;Acc:HGNC:17393] | 6357 | -0.032 | -0.2031 | No |
| 63 | CD74 | CD74 molecule [Source:HGNC Symbol;Acc:HGNC:1697] | 6374 | -0.033 | -0.2036 | No |
| 64 | KAT5 | lysine acetyltransferase 5 [Source:HGNC Symbol;Acc:HGNC:5275] | 6379 | -0.034 | -0.2033 | No |
| 65 | ANKRD1 | ankyrin repeat domain 1 [Source:HGNC Symbol;Acc:HGNC:15819] | 6416 | -0.037 | -0.2054 | No |
| 66 | PML | PML nuclear body scaffold [Source:HGNC Symbol;Acc:HGNC:9113] | 6421 | -0.037 | -0.2049 | No |
| 67 | CDK5RAP3 | CDK5 regulatory subunit associated protein 3 [Source:HGNC Symbol;Acc:HGNC:18673] | 6583 | -0.048 | -0.2166 | No |
| 68 | MYO6 | myosin VI [Source:HGNC Symbol;Acc:HGNC:7605] | 6816 | -0.064 | -0.2335 | No |
| 69 | TFAP4 | transcription factor AP-4 [Source:HGNC Symbol;Acc:HGNC:11745] | 6823 | -0.065 | -0.2326 | No |
| 70 | MAP2K6 | mitogen-activated protein kinase kinase 6 [Source:HGNC Symbol;Acc:HGNC:6846] | 7246 | -0.099 | -0.2637 | No |
| 71 | MDM2 | MDM2 proto-oncogene [Source:HGNC Symbol;Acc:HGNC:6973] | 7273 | -0.101 | -0.2637 | No |
| 72 | PLA2R1 | phospholipase A2 receptor 1 [Source:HGNC Symbol;Acc:HGNC:9042] | 7321 | -0.103 | -0.2653 | No |
| 73 | ATRX | ATRX chromatin remodeler [Source:HGNC Symbol;Acc:HGNC:886] | 7551 | -0.123 | -0.2808 | No |
| 74 | DYRK1A | dual specificity tyrosine phosphorylation regulated kinase 1A [Source:HGNC Symbol;Acc:HGNC:3091] | 7667 | -0.132 | -0.2871 | No |
| 75 | MUC1 | "mucin 1, cell surface associated [Source:HGNC Symbol;Acc:HGNC:7508]" | 7871 | -0.146 | -0.3001 | No |
| 76 | PRMT5 | protein arginine methyltransferase 5 [Source:HGNC Symbol;Acc:HGNC:10894] | 7904 | -0.148 | -0.2996 | No |
| 77 | RPS6KA6 | ribosomal protein S6 kinase A6 [Source:HGNC Symbol;Acc:HGNC:10435] | 8071 | -0.161 | -0.3093 | No |
| 78 | E2F2 | E2F transcription factor 2 [Source:HGNC Symbol;Acc:HGNC:3114] | 8090 | -0.162 | -0.3075 | No |
| 79 | PAXIP1 | PAX interacting protein 1 [Source:HGNC Symbol;Acc:HGNC:8624] | 8148 | -0.167 | -0.3086 | No |
| 80 | POU4F2 | POU class 4 homeobox 2 [Source:HGNC Symbol;Acc:HGNC:9219] | 8292 | -0.178 | -0.3162 | No |
| 81 | HEXIM1 | HEXIM P-TEFb complex subunit 1 [Source:HGNC Symbol;Acc:HGNC:24953] | 8293 | -0.178 | -0.3126 | No |
| 82 | TP53BP2 | tumor protein p53 binding protein 2 [Source:HGNC Symbol;Acc:HGNC:12000] | 8336 | -0.181 | -0.3122 | No |
| 83 | EDA2R | ectodysplasin A2 receptor [Source:HGNC Symbol;Acc:HGNC:17756] | 8364 | -0.183 | -0.3106 | No |
| 84 | KDM1A | lysine demethylase 1A [Source:HGNC Symbol;Acc:HGNC:29079] | 8608 | -0.202 | -0.3256 | No |
| 85 | NDRG1 | N-myc downstream regulated 1 [Source:HGNC Symbol;Acc:HGNC:7679] | 8688 | -0.208 | -0.3276 | No |
| 86 | SIRT1 | sirtuin 1 [Source:HGNC Symbol;Acc:HGNC:14929] | 8947 | -0.227 | -0.3432 | No |
| 87 | UBB | ubiquitin B [Source:HGNC Symbol;Acc:HGNC:12463] | 9142 | -0.243 | -0.3535 | No |
| 88 | NUAK1 | NUAK family kinase 1 [Source:HGNC Symbol;Acc:HGNC:14311] | 9190 | -0.247 | -0.3522 | No |
| 89 | RPL37 | ribosomal protein L37 [Source:HGNC Symbol;Acc:HGNC:10347] | 9265 | -0.253 | -0.3529 | No |
| 90 | SOX4 | SRY-box transcription factor 4 [Source:HGNC Symbol;Acc:HGNC:11200] | 9291 | -0.255 | -0.3497 | No |
| 91 | POU4F1 | POU class 4 homeobox 1 [Source:HGNC Symbol;Acc:HGNC:9218] | 9300 | -0.256 | -0.3451 | No |
| 92 | BAX | "BCL2 associated X, apoptosis regulator [Source:HGNC Symbol;Acc:HGNC:959]" | 9323 | -0.257 | -0.3416 | No |
| 93 | PLK2 | polo like kinase 2 [Source:HGNC Symbol;Acc:HGNC:19699] | 9482 | -0.273 | -0.3485 | No |
| 94 | SNAI2 | snail family transcriptional repressor 2 [Source:HGNC Symbol;Acc:HGNC:11094] | 9697 | -0.291 | -0.3594 | No |
| 95 | PLK3 | polo like kinase 3 [Source:HGNC Symbol;Acc:HGNC:2154] | 9698 | -0.291 | -0.3535 | No |
| 96 | C16orf72 | chromosome 16 open reading frame 72 [Source:HGNC Symbol;Acc:HGNC:30103] | 9902 | -0.312 | -0.3631 | Yes |
| 97 | TRIM24 | tripartite motif containing 24 [Source:HGNC Symbol;Acc:HGNC:11812] | 9927 | -0.314 | -0.3586 | Yes |
| 98 | USP10 | ubiquitin specific peptidase 10 [Source:HGNC Symbol;Acc:HGNC:12608] | 9955 | -0.317 | -0.3543 | Yes |
| 99 | PMAIP1 | phorbol-12-myristate-13-acetate-induced protein 1 [Source:HGNC Symbol;Acc:HGNC:9108] | 10084 | -0.329 | -0.3577 | Yes |
| 100 | RPL5 | ribosomal protein L5 [Source:HGNC Symbol;Acc:HGNC:10360] | 10201 | -0.341 | -0.3599 | Yes |
| 101 | BATF | basic leucine zipper ATF-like transcription factor [Source:HGNC Symbol;Acc:HGNC:958] | 10312 | -0.352 | -0.3614 | Yes |
| 102 | HIPK1 | homeodomain interacting protein kinase 1 [Source:HGNC Symbol;Acc:HGNC:19006] | 10391 | -0.360 | -0.3602 | Yes |
| 103 | CASP2 | caspase 2 [Source:HGNC Symbol;Acc:HGNC:1503] | 10506 | -0.374 | -0.3616 | Yes |
| 104 | RPS7 | ribosomal protein S7 [Source:HGNC Symbol;Acc:HGNC:10440] | 10608 | -0.384 | -0.3617 | Yes |
| 105 | IFI16 | interferon gamma inducible protein 16 [Source:HGNC Symbol;Acc:HGNC:5395] | 10614 | -0.385 | -0.3543 | Yes |
| 106 | PYHIN1 | pyrin and HIN domain family member 1 [Source:HGNC Symbol;Acc:HGNC:28894] | 10635 | -0.386 | -0.3481 | Yes |
| 107 | DDIT4 | DNA damage inducible transcript 4 [Source:HGNC Symbol;Acc:HGNC:24944] | 10818 | -0.407 | -0.3541 | Yes |
| 108 | RRS1 | ribosome biogenesis regulator 1 homolog [Source:HGNC Symbol;Acc:HGNC:17083] | 11045 | -0.437 | -0.3630 | Yes |
| 109 | MDM4 | MDM4 regulator of p53 [Source:HGNC Symbol;Acc:HGNC:6974] | 11047 | -0.437 | -0.3542 | Yes |
| 110 | EIF5A | eukaryotic translation initiation factor 5A [Source:HGNC Symbol;Acc:HGNC:3300] | 11127 | -0.446 | -0.3514 | Yes |
| 111 | MYBBP1A | MYB binding protein 1a [Source:HGNC Symbol;Acc:HGNC:7546] | 11188 | -0.456 | -0.3469 | Yes |
| 112 | ATR | ATR serine/threonine kinase [Source:HGNC Symbol;Acc:HGNC:882] | 11231 | -0.461 | -0.3408 | Yes |
| 113 | RPL11 | ribosomal protein L11 [Source:HGNC Symbol;Acc:HGNC:10301] | 11252 | -0.464 | -0.3330 | Yes |
| 114 | URB2 | URB2 ribosome biogenesis homolog [Source:HGNC Symbol;Acc:HGNC:28967] | 11291 | -0.470 | -0.3265 | Yes |
| 115 | FOXO3 | forkhead box O3 [Source:HGNC Symbol;Acc:HGNC:3821] | 11305 | -0.472 | -0.3179 | Yes |
| 116 | PAK1IP1 | PAK1 interacting protein 1 [Source:HGNC Symbol;Acc:HGNC:20882] | 11345 | -0.478 | -0.3113 | Yes |
| 117 | BCL3 | BCL3 transcription coactivator [Source:HGNC Symbol;Acc:HGNC:998] | 11431 | -0.493 | -0.3080 | Yes |
| 118 | CD44 | CD44 molecule (Indian blood group) [Source:HGNC Symbol;Acc:HGNC:1681] | 11439 | -0.494 | -0.2986 | Yes |
| 119 | STK11 | serine/threonine kinase 11 [Source:HGNC Symbol;Acc:HGNC:11389] | 11451 | -0.496 | -0.2894 | Yes |
| 120 | RPS20 | ribosomal protein S20 [Source:HGNC Symbol;Acc:HGNC:10405] | 11507 | -0.502 | -0.2836 | Yes |
| 121 | PPP1R13B | protein phosphatase 1 regulatory subunit 13B [Source:HGNC Symbol;Acc:HGNC:14950] | 11589 | -0.513 | -0.2795 | Yes |
| 122 | RPS15 | ribosomal protein S15 [Source:HGNC Symbol;Acc:HGNC:10388] | 11671 | -0.527 | -0.2752 | Yes |
| 123 | TWIST1 | twist family bHLH transcription factor 1 [Source:HGNC Symbol;Acc:HGNC:12428] | 11696 | -0.531 | -0.2663 | Yes |
| 124 | EP300 | E1A binding protein p300 [Source:HGNC Symbol;Acc:HGNC:3373] | 11741 | -0.540 | -0.2588 | Yes |
| 125 | RRN3 | "RRN3 homolog, RNA polymerase I transcription factor [Source:HGNC Symbol;Acc:HGNC:30346]" | 11762 | -0.543 | -0.2494 | Yes |
| 126 | CHD5 | chromodomain helicase DNA binding protein 5 [Source:HGNC Symbol;Acc:HGNC:16816] | 11776 | -0.545 | -0.2394 | Yes |
| 127 | MTOR | mechanistic target of rapamycin kinase [Source:HGNC Symbol;Acc:HGNC:3942] | 12047 | -0.605 | -0.2483 | Yes |
| 128 | MSX1 | msh homeobox 1 [Source:HGNC Symbol;Acc:HGNC:7391] | 12054 | -0.609 | -0.2365 | Yes |
| 129 | HIC1 | HIC ZBTB transcriptional repressor 1 [Source:HGNC Symbol;Acc:HGNC:4909] | 12118 | -0.624 | -0.2288 | Yes |
| 130 | AEN | apoptosis enhancing nuclease [Source:HGNC Symbol;Acc:HGNC:25722] | 12125 | -0.625 | -0.2166 | Yes |
| 131 | PIDD1 | p53-induced death domain protein 1 [Source:HGNC Symbol;Acc:HGNC:16491] | 12172 | -0.636 | -0.2073 | Yes |
| 132 | USP7 | ubiquitin specific peptidase 7 [Source:HGNC Symbol;Acc:HGNC:12630] | 12289 | -0.673 | -0.2028 | Yes |
| 133 | HNRNPK | heterogeneous nuclear ribonucleoprotein K [Source:HGNC Symbol;Acc:HGNC:5044] | 12341 | -0.688 | -0.1929 | Yes |
| 134 | SGK1 | serum/glucocorticoid regulated kinase 1 [Source:HGNC Symbol;Acc:HGNC:10810] | 12357 | -0.694 | -0.1800 | Yes |
| 135 | SNAI1 | snail family transcriptional repressor 1 [Source:HGNC Symbol;Acc:HGNC:11128] | 12365 | -0.696 | -0.1665 | Yes |
| 136 | NOP2 | NOP2 nucleolar protein [Source:HGNC Symbol;Acc:HGNC:7867] | 12372 | -0.699 | -0.1528 | Yes |
| 137 | DYRK3 | dual specificity tyrosine phosphorylation regulated kinase 3 [Source:HGNC Symbol;Acc:HGNC:3094] | 12379 | -0.702 | -0.1390 | Yes |
| 138 | RPL23 | ribosomal protein L23 [Source:HGNC Symbol;Acc:HGNC:10316] | 12558 | -0.777 | -0.1373 | Yes |
| 139 | BOP1 | BOP1 ribosomal biogenesis factor [Source:HGNC Symbol;Acc:HGNC:15519] | 12580 | -0.785 | -0.1230 | Yes |
| 140 | USP2 | ubiquitin specific peptidase 2 [Source:HGNC Symbol;Acc:HGNC:12618] | 12648 | -0.824 | -0.1116 | Yes |
| 141 | CDKN1A | cyclin dependent kinase inhibitor 1A [Source:HGNC Symbol;Acc:HGNC:1784] | 12727 | -0.890 | -0.0997 | Yes |
| 142 | MYC | "MYC proto-oncogene, bHLH transcription factor [Source:HGNC Symbol;Acc:HGNC:7553]" | 12801 | -0.970 | -0.0858 | Yes |
| 143 | DDX5 | DEAD-box helicase 5 [Source:HGNC Symbol;Acc:HGNC:2746] | 12834 | -1.038 | -0.0673 | Yes |
| 144 | HIPK2 | homeodomain interacting protein kinase 2 [Source:HGNC Symbol;Acc:HGNC:14402] | 12842 | -1.045 | -0.0467 | Yes |
| 145 | TAF1 | TATA-box binding protein associated factor 1 [Source:HGNC Symbol;Acc:HGNC:11535] | 12881 | -1.192 | -0.0255 | Yes |
| 146 | GTSE1 | G2 and S-phase expressed 1 [Source:HGNC Symbol;Acc:HGNC:13698] | 12894 | -1.332 | 0.0005 | Yes |
Table: GSEA details [plain text format]

  

Fig 2: GOBP\_SIGNAL\_TRANSDUCTION\_BY\_P53\_CLASS\_MEDIATOR      
 Blue-Pink O' Gram in the Space of the Analyzed GeneSet

  

Fig 3: GOBP\_SIGNAL\_TRANSDUCTION\_BY\_P53\_CLASS\_MEDIATOR: Random ES distribution      
 Gene set null distribution of ES for **GOBP\_SIGNAL\_TRANSDUCTION\_BY\_P53\_CLASS\_MEDIATOR**
